# Computerized tongue image analysis for non-invasive disease screening: a review

**DOI:** 10.1186/s13020-025-01242-7

**Published:** 2025-11-21

**Authors:** Huangbo Lin, Zhihan Ning, Chenglong Zhang, Shaoyang Men, David Zhang

**Affiliations:** 1 School of Medicial Information Engineering, Guangzhou University of Chinese Medicine, 232 Waihuandong Road, Guangzhou, 510006 Guangdong China; 2 School of Data Science, The Chinese University of Hong Kong, Shenzhen, 2001 Longxiang Boulevard, Shenzhen, 518172 Guangdong China; 3 School of Science and Engineering, The Chinese University of Hong Kong, Shenzhen, 2001 Longxiang Boulevard, Shenzhen, 518172 Guangdong China

**Keywords:** Computerized tongue image analysis, Non-invasive digital biomarker, Remote healthcare, Health monitoring, Survey

## Abstract

The characteristics of the tongue surface and sublingual vein patterns provide valuable insights into an individual’s health status and have long served as the cornerstone of traditional tongue diagnosis. As a non-invasive digital biomarker, tongue imaging has recently gained attention as a promising modality for capturing internal physiological and pathological variations, with the potential to support remote healthcare delivery and continuous health monitoring. Nevertheless, conventional practice remains highly dependent on subjective clinical judgment, which often introduces variability in diagnostic accuracy and therapeutic decision-making. To mitigate these limitations, computerized tongue image analysis (CTIA) has been developed to enhance objectivity, reproducibility, and consistency. This review proposes a structured taxonomy of CTIA, encompassing the essential stages of image acquisition, preprocessing, dataset construction, feature extraction, and disease detection. By systematically synthesizing advances across these stages, we delineate key challenges and outline potential solutions, particularly regarding data standardization and feature quantification. The taxonomy is intended to provide a coherent framework that may contribute to improving diagnostic precision and reliability, thereby informing the gradual clinical integration of tongue imaging as a supportive tool for non-invasive disease screening.

## Introduction

The tongue is the only organ capable of protruding from the body [1], functioning as an essential component of physiological structures while simultaneously serving as a critical visual indicator of a wide range of physiological and pathological conditions. Its external visibility allows for a nuanced examination and interpretation of complex bodily states, making it a valuable diagnostic tool in clinical practice. The characteristics of the tongue body, coating, and sublingual veins, along with associated pathological anomalies, are primarily shaped by the integrated anatomical and physiological interactions among the tongue’s neural, muscular, vascular, and papillary structures [2, 3]. These underlying mechanisms give rise to a spectrum of diagnostic features, including variations in color, texture, geometric configuration, and temporal dynamics, alongside key physiological indicators such as morphology and motility. The measurement of digital biomarkers is inextricably linked to the advancement of digital health technologies, catalyzing transformative changes in the healthcare sector [4]. The tongue image, as a key non-invasive digital biomarker, provides indispensable support for remote healthcare and health monitoring. Digital biomarkers, particularly those used for remotely monitoring symptoms, possess the potential to fundamentally transform the methodologies of outcome assessment in future disease diagnosis [5].

**Fig. 1:**
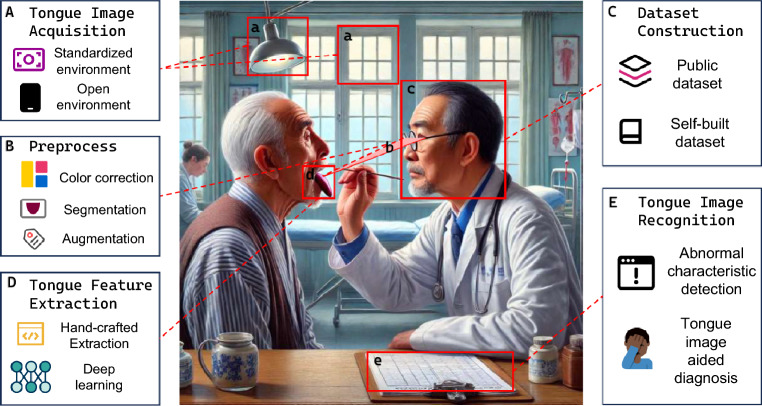
Relationship between traditional TD and the CTIA. The middle part of this figure showcasing the medical examination example is generated by GPT-4 (OpenAI). To objectify traditional TD, CTIA (**A**) employs tongue imaging devices in either a standardized environment or an open environment to collect tongue images, (**B**) preprocesses collected images by conducting color correlation, tongue segmentation, and data augmentation, (**C**) constructs dataset, (**D**) feature extraction to describe various tongue characteristics, and (**E**) trains diagnosis model via machine learning approaches for tongue image recognition and disease detection

For example, the formation of tongue color is closely related to changes in tissue blood flow, dietary factors, dehydration, anemia, fungal growth, niacin, folic acid, vitamins, nuclear factor-kappa B pathway activation, and impaired microvascular circulation in the tongue [6–9]. The intrinsic membrane of the tongue is rich in blood vessels, lymphatic vessels, nerves, and glandular organs, which contribute to variations in the geometry and texture of the tongue [10, 11]. The tongue coating develops from the differentiation of dorsal lingual mucosal tissues, involving the balanced regulation of proliferation, differentiation, apoptosis, and shedding of epithelial cells. Differential expression of apoptosis-related genes is a key molecular mechanism regulating tongue coating formation [12, 13]. Additionally, the microbiota of the tongue coating influences the gut microbiota, primarily through mechanisms such as oral microbes colonizing the gut via the bloodstream or digestive tract, or the transfer of microbial metabolites via the immune system [14] Fig.  1.

Through the extensive pathological information conveyed by the tongue, tongue diagnosis (TD) has become an essential non-invasive modality for early disease screening, prevention, and prognostic assessment. Due to its accessibility, real-time applicability, high efficiency, lack of adverse effects, and pain-free nature, this diagnostic approach has attracted considerable international recognition [3, 15]. Numerous studies [16–18] have demonstrated a strong correlation between tongue images and a range of serious and chronic conditions, infectious diseases, and suboptimal health states. However, as an alternative and auxiliary diagnostic approach, TD is susceptible to variability stemming from subjective factors, including the clinician’s expertise, patient cooperation, and environmental conditions, leading to inconsistencies in clinical outcomes. These limitations have constrained the further advancement and widespread adoption of TD methodologies.

To address the challenges associated with traditional TD, computerized tongue image analysis (CTIA) methods have been thoroughly studied to make the TD process objective, convenient, and reproducible. Typical CTIA approaches mainly focus on developing innovative data acquisition devices and employing computer vision techniques to enhance the perceptual and analytical processes of traditional TD. With the recent flourishing of digital sensing and machine learning (ML) techniques, CTIA approaches have garnered significant international interest and have become an increasingly popular research topic, attracting a growing number of researchers to the field [15, 17, 18]. The study of CTIA enables more precise and fine-grained characterization of tongue features.

**Fig. 2:**
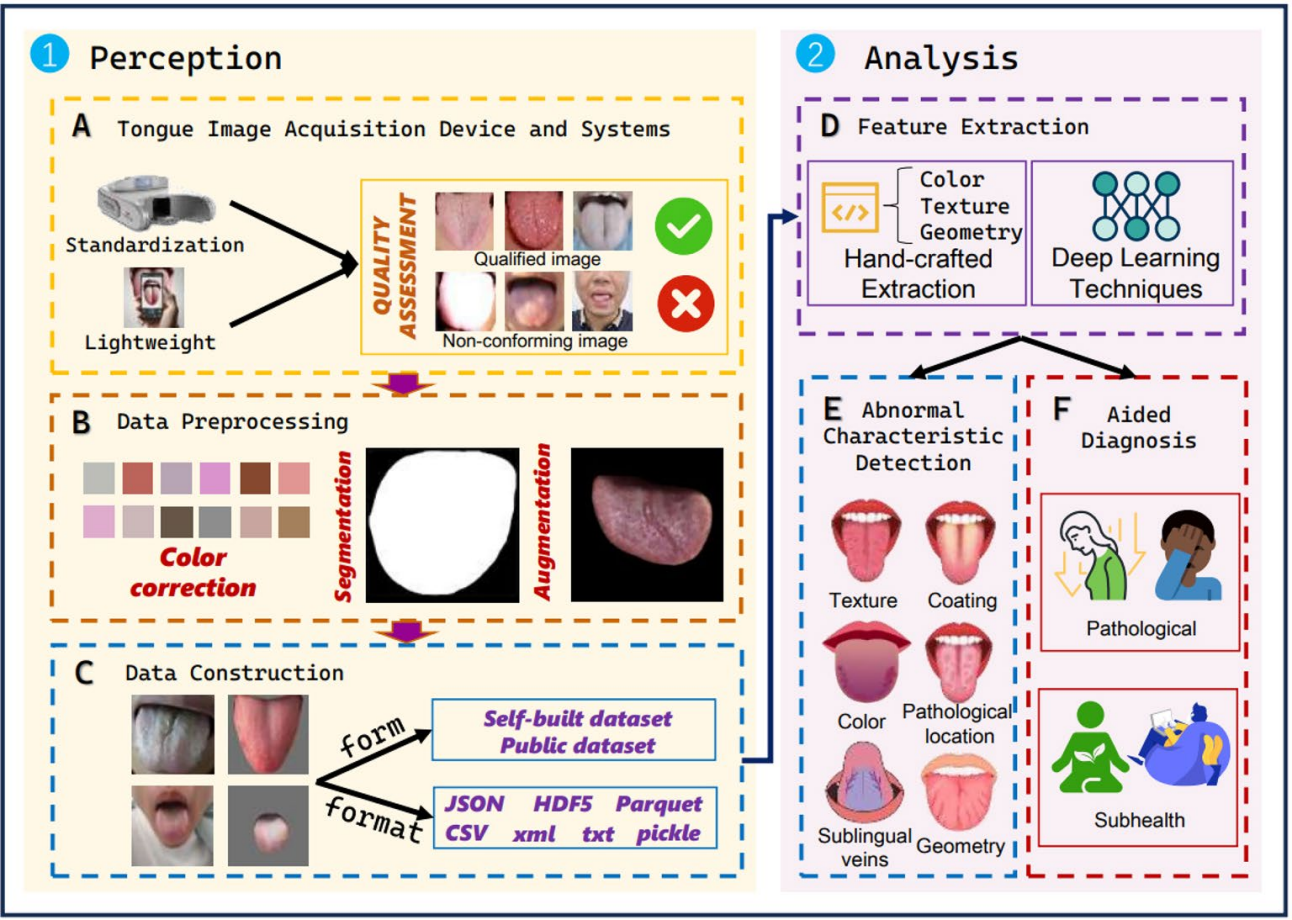
An overview of the CTIA. This article is mainly divided into two sections: perception and analysis. The three topics under perception include: (**A**) Tongue Image Acquisition Device and Systems, which discusses the advantages and disadvantages of the standard environment and open environment acquisition strategies, as well as some system solutions; (**B**) Data Preprocessing, which covers some common processing methods, including color correction, segmentation, and augmentation; (**C**) Data Construction, which considers the construction of self-built and public datasets (Some details can be found in Appendix B). The three topics under analysis include: (**D**) two approaches to feature extraction; (**E**) categories of abnormal characteristic detection; and (**F**) methods for assisted diagnosis. On the right side of the diagram is a brief flowchart representation of the process

CTIA mainly includes five essential stages, i.e., tongue image acquisition, data preprocessing, dataset construction, feature extraction, and image recognition, as shown in Fig. 2. Extensive studies have been conducted regarding CTIA with the advancements of recent sensing and ML techniques. While some existing reviews and surveys [19–22] have traced the advancements in this area, none of them have presented a structured taxonomy and systematic analysis covering the whole procedure of CTIA. In this paper, we systematically study the recent advancements of CTIA and provide a well-organized taxonomy, in-depth analysis, and actionable future perspectives for tongue image *perception* and *analysis*. In this review, we conducted a comprehensive review grounded in a novel taxonomy, with the aim of bridging the gap between clinical interpretation and technical analysis. Through this synthesis, we not only offer a deep exploration of the current landscape but also highlight the key challenges that lie ahead Table 1, 2.

**Table 1:** Representative cases of the whole process of CTIA

**Ref.**	Device		Preprocessing				Feature		Task			Method			Data source			**DataVolume**	**Label**
	OE	SE	Ca.	Se.	Au.	De.	Hf.	Df.	Ac.	Di.	Mu.	TM	ML	DL	Pr.	Or.	Pu.		
[23]	**N**	*Y*	**N**	**N**	**N**	**N**	*Y*	**N**	**N**	*Y*	*Y*	*Y*	**N**	**N**	*Y*	*Y*	**N**	None	Appendicitis, pancreatitis
[24]	**N**	*Y*	**N**	*Y*	**N**	*Y*	*Y*	**N**	**N**	**N**	**N**	*Y*	**N**	**N**	*Y*	*Y*	**N**	150	Sublingual vein colors
[25]	**N**	*Y*	**N**	**N**	**N**	**N**	*Y*	**N**	*Y*	**N**	**N**	*Y*	**N**	**N**	**N**	*Y*	**N**	174	Live, fake
[26]	**N**	*Y*	*Y*	**N**	**N**	**N**	*Y*	**N**	*Y*	**N**	*Y*	*Y*	*Y*	**N**	*Y*	*Y*	**N**	(362)	Tongue shape
[27]	**N**	*Y*	**N**	**N**	**N**	*Y*	*Y*	**N**	**N**	**N**	**N**	*Y*	**N**	**N**	*Y*	**N**	**N**	(36000)	Sublingual vein
[28]	**N**	*Y*	*Y*	**N**	**N**	*Y*	*Y*	**N**	*Y*	*Y*	*Y*	*Y*	*Y*	**N**	*Y*	**N**	**N**	5222 (9000)	Disease, tongue color
[29]	**N**	*Y*	**N**	**N**	**N**	**N**	*Y*	**N**	**N**	*Y*	*Y*	*Y*	**N**	**N**	*Y*	**N**	**N**	504	DM, IGR
[30]	**N**	*Y*	**N**	*Y*	**N**	*Y*	*Y*	**N**	**N**	*Y*	*Y*	*Y*	*Y*	**N**	*Y*	**N**	**N**	None	Thyroid, ulcer
[31]	*Y*	**N**	*Y*	**N**	**N**	**N**	*Y*	**N**	**N**	**N**	*Y*	*Y*	*Y*	**N**	**N**	*Y*	**N**	246 (726)	Lighting conditions, tongue fur
[32]	**N**	*Y*	**N**	*Y*	*Y*	**N**	**N**	*Y*	*Y*	**N**	*Y*	**N**	**N**	*Y*	*Y*	**N**	**N**	1858	Tongue coating state(6)
[33]	**N**	*Y*	**N**	**N**	*Y*	**N**	**N**	*Y*	**N**	*Y*	**N**	**N**	**N**	*Y*	**N**	**N**	**N**	(1570)	Healthy, patient
[34]	**N**	*Y*	*Y*	*Y*	**N**	**N**	*Y*	**N**	**N**	*Y*	**N**	**N**	*Y*	**N**	*Y*	**N**	**N**	1778	NAFLD, non-NAFLD
[35]	**N**	**N**	**N**	**N**	**N**	**N**	*Y*	**N**	**N**	*Y*	**N**	*Y*	*Y*	**N**	*Y*	**N**	**N**	10602	Syndrome types of primary liver cancer(8)
[36]	**N**	*Y*	**N**	*Y*	**N**	**N**	*Y*	**N**	**N**	*Y*	*Y*	**N**	*Y*	**N**	*Y*	**N**	**N**	903	Disease(5)
[37]	*Y*	*Y*	*Y*	*Y*	**N**	*Y*	*Y*	**N**	*Y*	**N**	*Y*	**N**	*Y*	*Y*	*Y*	**N**	**N**	(10333)	Disease location
[38]	**N**	*Y*	**N**	*Y*	*Y*	**N**	**N**	*Y*	*Y*	**N**	*Y*	**N**	**N**	*Y*	*Y*	**N**	*Y*	(1703)	Anomaly characteristic
[10]	**N**	**N**	**N**	*Y*	**N**	**N**	**N**	*Y*	*Y*	**N**	*Y*	**N**	**N**	*Y*	**N**	**N**	*Y*	(1151)	thin, regular, and enlarged tongues
[39]	**N**	*Y*	*Y*	*Y*	**N**	**N**	*Y*	**N**	**N**	*Y*	**N**	*Y*	**N**	**N**	*Y*	**N**	**N**	385	Acute ischemic stroke
[40]	**N**	*Y*	*Y*	*Y*	**N**	**N**	*Y*	*Y*	**N**	*Y*	*Y*	**N**	*Y*	*Y*	*Y*	**N**	**N**	(440)	Fatty liver
[17]	**N**	*Y*	*Y*	*Y*	*Y*	*Y*	*Y*	*Y*	**N**	*Y*	**N**	**N**	**N**	*Y*	*Y*	**N**	**N**	732	Diabetes
[41]	**N**	**N**	**N**	*Y*	**N**	**N**	**N**	*Y*	**N**	*Y*	**N**	**N**	**N**	*Y*	**N**	**N**	**N**	4295	Gastric cancer
[42]	**N**	*Y*	**N**	**N**	**N**	**N**	*Y*	**N**	**N**	*Y*	**N**	**N**	*Y*	**N**	*Y*	**N**	**N**	3689	DM, CKD, BC, CG

(a) OE is open environment; (b) SE is standardized environment;

(c) Ca. is color/shape calibration; (d) Se. is segmentation;

(e) Au. is augmentation; (f) De. is denoising; (g) Hf. is handcrafted features;

(h) Df. is deep features; (i) Ac. is anomaly characteristic detection;

(j) Di. is disease diagnosis; (k) Mu. is multi-label and multi-modal;

(l) TM is traditional method; (m) ML is machine learning;

(n) DL is deep learning; (o) Pr. is professional venues; (p) Or. is ordinary places;

(q) Pu. is public datasets; (r) Data volume: number of participants (number of images);

(s) Task: if all tasks are not marked, they are segment tasks;

(t) Y and N**:** respectively represent having or not having

## Tongue image perception

### Tongue image acquisition device and systems

In the process of CTIA through perception and analysis, capturing and representing tongue images digitally is an essential step, whether using computerized medical techniques or leveraging the tongue’s physiological characteristics for biometric identification [48]. Developing high-quality and consistent tongue imaging systems is crucial for advancing and popularizing computerized TD [49]. The mainstream devices used for capturing tongue features can be categorized into two types: standardized acquisition devices and open acquisition devices.

#### Standardized acquisition devices and systems

Different light sources, varying in color temperature, illuminance, and color rendering index, produce distinct color presentations on illuminated objects. To achieve consistent color fidelity, true-to-life imaging, and reproducibility, standardized imaging devices have been developed. Common color standards include RGB, HSV, Lab, YCbCr, YIQ, CIE Luv, and CMYK [50]. Among lighting standards, D65 [18] and D50 [51] are widely used; D65, which approximates daylight, is particularly favored for imaging and color matching applications. Common light sources include fluorescent lamps, halogen lamps, metal halide lamps, and LED lamps. Fluorescent and halogen lamps offer good color rendering performance, while metal halide lamps excel in color stability. However, metal halide lamps tend to be larger in size and have poorer heat dissipation. LED lamps are favored for their low power consumption and spectral tuning flexibility, making them suitable for various imaging needs, although they may have a relatively lower color rendering index. In imaging devices, three-CCD cameras are widely used for tongue image acquisition, as their independent channel structure enhances color accuracy and clarity. In contrast, single-CCD cameras, while more cost-effective, exhibit deficiencies in color fidelity and resolution. To minimize motion blur, video cameras are often preferred to ensure stability and consistency during the acquisition process. Further technological advancements include the integration of spectral filters to enhance color consistency under various light sources, as well as the incorporation of mirror designs within the imaging pathway to capture tongue images from multiple angles. These optimizations significantly enhance the versatility of the equipment and improve the diagnostic reliability of the images obtained. In terms of temporal analysis, the feature analysis of single long-duration video streams [52–54] and sequential image capture [55–57] have further advanced research on tongue imagery.

At the same time, to build a system workflow integrating tongue image acquisition, detection, segmentation, feature extraction, and disease diagnosis, a significant amount of systematic work has provided technical support for research in non-engineering fields [58]. In particular, the CTIA [59], as an integrated online system workflow solution, demonstrates a clear advantage in image consistency and reliability. It effectively overcomes external interference factors such as lighting conditions and capture angles, significantly improving the objectivity and reproducibility of diagnoses.

#### Open acquisition protocol

To overcome the limitations of standardized equipment’s mobility in real-world environments, TD applications have been developed for smartphones and tablets. These applications enable data collection, tongue image capture, and automatic analysis in open environments, providing preliminary support for health assessments [60]. However, practical applications still face variability issues arising from differences in smartphone models, capture methods, and inconsistent lighting conditions [61]. Lightweight solutions often come at the cost of precision, in exchange for real-time feedback.

#### Multi-media sensing

In addition to conventional methods using digital cameras for image capture, various approaches are being explored to expand the boundaries of TD research. These methods aim to uncover additional dimensions of information that are not typically captured by digital cameras, thereby advancing the modernization of comprehensive TD studies.

*Hyperspectral imaging*. Reflectance data across multiple spectral bands allows for a more detailed analysis of the tongue compared to conventional color images [62]. This rich spectral information facilitates more accurate and sensitive characterizations and classifications of the tongue surface and coating, while also enabling the extraction of advanced semantic features [63, 64]. Early studies [62] employed spectral angle mapping to analyze tongue color, leveraging spectral data to delineate significant material regions and tongue coatings. However, manual feature extraction has not fully harnessed the potential of hyperspectral imagery. DL has become widely adopted for hyperspectral image classification [63] due to its capacity to autonomously extract pertinent features from complex, high-dimensional data. It has also demonstrated the ability to digitally map the tongue’s color space and coatings [65].

*Three-dimensional imaging*. Using multi-angle data acquisition and advanced image reconstruction algorithms, three-dimensional imaging enables the creation of a 3D model of the tongue. This approach not only captures the appearance and morphology of the tongue but also provides detailed spatial structural information.

*Infrared thermography imaging*. This media is a non-contact measurement technique that detects infrared radiation emitted by an object to obtain temperature distribution data. Applied to tongue imaging, it reveals temperature variations on the tongue’s surface, which are closely linked to physiological processes such as metabolic status and inflammatory responses. This technology is frequently used for assessing sublingual vein width and segmentation tasks. Relevant studies [66, 67] have utilized the characteristics of near-infrared sublingual images to effectively isolate sublingual veins from the surrounding tongue tissue and precisely trace their boundaries.

**Fig. 3:**
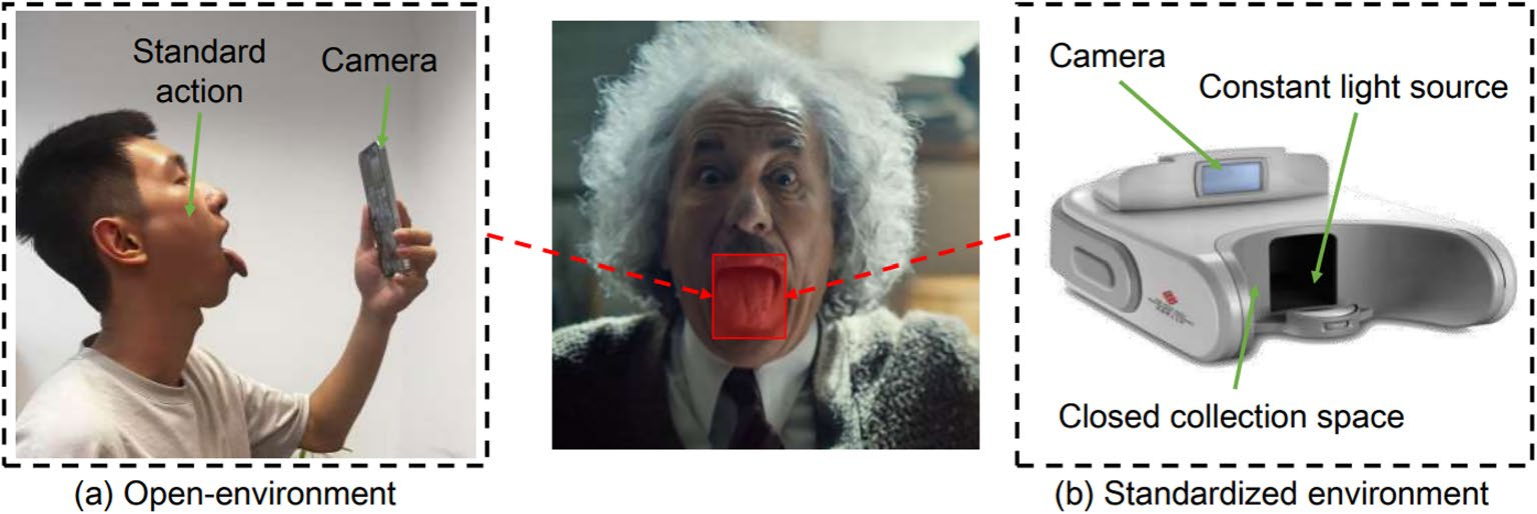
Demonstration of open scene and standardized scene acquisition methods

*Ultrasound sensing*. As a non-invasive and clinically safe imaging method, ultrasound techniques leverages high-frequency sound wave reflections to observe internal biological structures in real-time. In TD, ultrasound enables dynamic monitoring of tongue movement and morphological changes, offering valuable physiological insights. Some studies [68, 69] have utilized real-time ultrasound video data to design a novel convolutional neural network (CNN) [70] inspired by human peripheral vision capabilities, specifically for the task of tongue contour tracking Fig.   3.

### Data preprocessing

The raw data obtained from tongue imaging are often unsuitable for immediate analysis due to various factors stemming from both environmental conditions and patient-specific influences. These issues may include non-tongue-related images, stained coating, mirror-like haziness, incomplete tongue display, partial extension, foreign objects on the tongue, abnormal postures, excessive camera distance, underexposure or overexposure, lighting drift and reflections, low resolution, artifacts, and image blurring. Additionally, interference from surrounding facial features, such as lips, teeth, and the upper jaw, can further compromise classification accuracy [71, 72].

To mitigate these challenges, several advanced preprocessing methods have been proposed to solve the data quality issues in both standardized and open environments, which are crucial for subsequent analysis results. In addition to manual screening tasks, such as removing non-sequential duplicate images, eliminating images with foreign objects or stained fur on the tongue, and traditional image processing techniques like cropping, resizing, and denoising, methods such as color calibration, tongue and sublingual vein segmentation, fur separation, and data augmentation are widely employed in the reconstruction of high-quality data. To facilitate image evaluation, some deep learning-based automated methods [71, 73] have been applied in image quality assessment (IQA) research to automatically reject substandard tongue images. The final step in data preprocessing involves tasks such as labeling classification features (binary and multi-class), bounding boxes, segmentation masks, and disease-related labels.

#### Color correction

Even for the same tongue, digital tongue images captured under different lighting conditions, using various cameras, and displayed on different monitors can result in inconsistent color appearances, potentially leading to varying diagnostic outcomes. The clinical significance of color is influenced by differences in light sources, camera technologies, and human perception. In color correction tasks, models like Lab, RGB, HSI, and HSV color spaces [45, 74] facilitate detailed color extraction, morphological analysis, and segmentation of tongue images, enabling a more comprehensive and multidimensional representation of tongue-related information. However, smartphone-based image acquisition poses two key challenges. Initially, variations in device specifications and manufacturers’ camera calibration methods frequently result in color discrepancies between the captured tongue images and their real-world appearance. Additionally, even when identical image processing algorithms are applied, subtle inconsistencies may emerge in the digital outputs across different tongue image acquisition devices.

A standardized environment typically refers to a controlled setting, where physical color charts play a crucial role in color calibration. The most commonly used color chart is the Munsell ColorChecker (MSCC) [75], which is considered the standard in the field of color calibration. This chart consists of 24 color patches, including 6 grayscale and 18 colors that mimic natural objects, which are used to calibrate all visible colors. However, the colors associated with the tongue are primarily red, reddish, or yellowish, and are confined to a relatively narrow range. To address the issue of standardizing tongue color data and better align with tongue color research, a new design for a tongue-specific ColorChecker has been proposed. This involves the use of color normalization clustering [76] and fuzzy C-means (FCM) clustering algorithms [77]. Furthermore, a method has been developed for a detailed explanation of the color chart design, where the selected Munsell ColorChecker’s hue-lightness-chroma (HLC) data is converted into CIE xyY, then transformed into CIELAB and sRGB values. This approach successfully integrates color, texture, and moisture into a computational tongue image simulation model [78].

Beyond conventional physical processing methods, a range of advanced techniques have emerged for color correction aimed at enhancing color calibration accuracy. These methodologies encompass white balance-based corrections, polynomial regression models, support vector regression (SVR) algorithms, neural network mappings, and finite-dimensional reflection models. Given the particularity of tongue images, methods such as the Two-Phase Deep Color Correction Network (TDCCN) [60], SA-GA-BP neural networks [79], and ICC profile correction [80] have been applied to achieve more precise color correction, particularly in the context of similar color comparisons. Furthermore, smartphone-based color correction solutions adaptable to multiple environments have provided convenience for non-engineering researchers [51].

#### Tongue segmentation

Segmentation tasks primarily encompass tongue body segmentation, sublingual vein segmentation, and tongue coating segmentation. Once the initial object contour mistakenly includes false boundary edges from surrounding tissues, it becomes challenging for the contour to converge accurately to the true boundary of the tongue body [81]. Segmentation algorithms have evolved significantly, transitioning from traditional image processing techniques to those based on DL.

*Traditional methods*. Include color thresholding [82], edge detection [83], active contour models (ACM) [84], and graph-based approaches [61]. While traditional methods provide strong interpretability and intuitive appeal, they often suffer from significant limitations when confronted with factors such as lighting variations, uneven textures, and scale changes. These challenges hinder their performance in complex or real-world applications. Although these methods rely on manually designed features and assumptions, making them highly interpretable, they are inherently vulnerable to noise and variations in the data. In dynamic and diverse environments, their discriminative capacity is often restricted, and their robustness and generalization ability are relatively weak, which compromises their ability to maintain consistent performance, particularly when dealing with complex or highly variable data.

*DL methods*. With the advancement of artificial intelligence, DL approaches have achieved superior discriminative capabilities, albeit at the expense of interpretability due to their black-box nature. Key DL strategies for segmentation include supervised methods, represented by U-Net [85, 86] and DeepLab [87], and unsupervised approaches, such as those based on Generative Adversarial Network (GAN) [88]. Nevertheless, while supervised learning methods are dependent on substantial labeled datasets, unsupervised approaches often exhibit heightened sensitivity to model configurations and parameter adjustments. Consequently, semi-supervised learning [89] has emerged as a promising alternative to enhance both the accuracy and generalization capabilities in tongue segmentation tasks. In parallel, lightweight networks such as the MobileNet series [90] have been proposed to facilitate the deployment of segmentation algorithms on mobile devices. This approach achieves accurate tongue image segmentation results while maintaining minimal model parameters and reduced computational complexity.

*Sublingual vein segmentation*. The segmentation of sublingual veins presents several significant challenges, including variations in image exposure, complex backgrounds, and the inherent connectivity of the veins. To address these issues, methods based on Generative Adversarial Networks (GANs) [91] have been widely adopted. In addition, the identification of small venous branches is crucial. A two-stage semantic segmentation approach has been proposed [92], which minimizes the loss of spatial feature information and captures multi-scale details, thereby improving the accuracy of fine vein branch recognition. This approach provides more precise data support for subsequent image analysis and diagnostic tasks. Furthermore, the complex relationship between the sublingual veins and the tongue body, as well as the enhancement of segmentation stability under varying lighting conditions, remain key challenges that need to be addressed in this field. The integration of Multi-task Learning (MTL) and Self-supervised Learning (SSL) holds promise for improving the robustness and generalization capacity of models in diverse and complex environments.

*Tongue coating segmentation*. Unlike tongue body segmentation, tongue coating segmentation presents greater challenges. It is worth noting that research on coating separation primarily focuses on methods such as dual-light source acquisition, traditional chromaticity thresholding [93], K-means-based machine learning, and deep learning approaches centered around U-net and attention mechanisms [94].

## Tongue image analysis

### Feature extraction

Once the dataset has been constructed, the next step is to develop the relevant feature space. In pattern recognition, feature extraction is a critical step. Traditional methods include handcrafted features and statistical features. Handcrafted features are defined based on human expertise, utilizing domain knowledge and statistical metrics to capture key characteristics within the data. These typically encompass aspects such as color, texture, and geometric features.

*Hand-crafted extraction*. Color feature extraction can be performed in various color spaces, including RGB, HSV, CIEYxy, CIELUV, and CIELAB. It is important to note that the HSV space exhibits discontinuity in hue values around red, making this approach sensitive to noise [50], particularly since the primary color of the tongue is red, which can affect the extraction and analysis results. Local texture features arise from regular variations in pixel color and brightness. When there is varying repetition of local texture information, it contributes to global texture information. The main methods for extracting these features include the Gray-Level Co-occurrence Matrix (GLCM), Local Binary Pattern (LBP), autoregressive texture models, and wavelet transformations. Image geometric feature extraction typically involves two methods: edge-based extraction and feature point-based descriptor operators. In practice, color, texture, and geometric features are usually considered together [18, 36].

*DL techniques*. Through an end-to-end learning framework, complex feature representations are autonomously extracted from raw data. By applying a variety of transformations and mappings, these approaches effectively capture high-dimensional features and latent representation, leading to a substantial improvement in performance on specific tasks. This not only improves model efficacy but also reduces computational costs and enhances the interpretability of the data, thereby bolstering the generalization capabilities of the model. As a result, DL models are better equipped to identify subtle patterns in tongue images that are pertinent to disease diagnosis. Deep features typically encompass rich semantic information, enabling their statistical representations to effectively capture both intra-class and inter-class variations [95–97]. Currently, widely utilized approaches for deep feature extraction include ResNet [73], VGG [98], Vector Quantized Variational Autoencoders (VQ-VAE) [17]and Wide Line Detector (WLDF) [99], among others.

*Fusion strategy*. The understanding of features at different levels significantly impacts model performance. Shallow features tend to have higher resolution, containing more spatial and detailed information, while deep features carry stronger semantic meaning. Some studies [100, 101] have explored the fusion of shallow and deep features, which can be categorized into early fusion and late fusion based on the order of integration and prediction. This dual strategy offers a more comprehensive and balanced analysis, allowing researchers to leverage the complementary advantages of both methods. Such fusion not only enhances the overall assessment of tongue image data but also contributes to improving the accuracy and reliability of clinical diagnoses.

**Table 2:** Feature extraction scheme example

Category	Refs	Methods
Color	[102]	By analyzing the reflectance of each pixel across 120 contiguous spectral bands, a high-dimensional spectral feature vector is constructed to represent the spectral information of the image.
	[36]	Color histogram and color moments are calculated to capture the distribution and statistical properties of the RGB channels.
	[42]	The images are mapped to a common shape, and Principal Component Analysis (PCA) is applied to extract 40-dimensional color-aligned features. The images are then converted to the CIELAB color space, where pixels are assigned to 12 predefined color centroids. The proportion of pixels assigned to each centroid is used to extract 96-dimensional color gamut features.
Geometry and texture	[26]	Length-based features include the aspect ratio, eccentricity, and radial line ratio, which describe the global and local length characteristics of the tongue shape. Area-based features include the total area ratio, triangular area ratio, and upper-lower area ratio, which describe the area characteristics of the tongue shape. Angle-based features include the average angle between the tongue sides and the horizontal line, which describes the angular characteristics of the tongue shape.
	[36]	Texture features are extracted using LBP and Gabor filters. Geometric features, such as length, width, area, and various shape descriptors, are also defined to characterize the shape and size of the sublingual vein.
	[42]	The Gabor filter responses of the aligned tongue images are used to extract 40-dimensional texture-aligned features. Gabor filtering is applied to the grayscale tongue images, and the maximum response is utilized to extract 8-dimensional Gabor texture features. Additionally, the Fast Fourier Transform (FFT) is applied to the tongue images to extract 20-dimensional geometric features.
Latent feature	[33]	The image is divided into five regions, with particular emphasis on the central region (Region 5) due to its richer information. Feature vectors are extracted using a two-layer artificial neural network (ANN), and the optimal network architecture and layers are selected based on information theory and mutual information to maximize class separability.
	[36]	Stacked sparse autoencoder (SSAE) is used to extract a latent representation from the sublingual vein region.
	[101]	Deep features were extracted from tongue images using the ResNet-50 DL model.
	[103]	Extraction of 4096-Dimensional Eigenvectors Using VGG Network.
Hybird	[37]	Stochastic Region Pooling (SRP) is used to extract local region features from tongue images, with the Intra-Imaging Channel Attention (InI) mechanism modeling and weighting these features to enable accurate disease localization.
	[30]	Using the tissue zone unsupervised technique, twelve features were extracted, including Root Mean Square (RMS), contrast, entropy, homogeneity, color analysis, cracked and smoothness analysis, size analysis, and coating analysis.
	[25]	The article proposes algorithms to extract geometric, crack, texture, squirm features, and a fusion of physiological static and squirm features.

### Anomaly characteristic detection

Practitioners document abnormal characteristics in tongue images as key indicators for disease diagnosis. These observations typically focus on five main aspects: tongue color, shape and texture, coating, sublingual veins, and pathological location. Please note that this concept should be distinguished from the general term “anomaly detection”, which refers to “the identification of patterns in data that do not conform to expected behavior or a standard pattern”, and is commonly used in fields such as fraud detection, network security, and system monitoring to identify unusual or rare events [104].

*Color*. Color is one of the primary features perceived by the human visual system, playing a crucial role in assessing tongue images [105–107]. The morphological variations of the lingual papillae are closely related to tongue color. Generally, it is rare for a single color to appear in isolation. In most cases, multiple colors are blended together. Sometimes, there is a gradation of shades or a clear division of different colors, where boundaries may either overlap or remain distinctly defined.

Tongue color analysis was one of the earliest and most extensively studied areas in the CTIA. Researchers primarily explore the variation in color attributes by constructing color feature vectors and defining the tongue color space [108, 109]. Notably, selecting a color gamut closely aligned with tongue color has proven crucial for accurate analysis. With the recent advancements in DL, these technologies have gradually been applied to tongue color analysis, enabling the extraction of high-level feature representations from images with high background similarity [110, 111].

*Geometry and texture*. The intrinsic membrane of the tongue is rich in blood vessels, lymphatic vessels, nerves, and glandular organs, which are related to the geometry and texture of the tongue [10, 11]. Most studies on feature recognition remain at the level of coarse-grained classification, without further exploration or refinement. In clinical research, quantifiable indicators such as the shape, location, number, and severity of cracks can establish a quantitative mapping relationship with diseases such as Down syndrome [112, 113], psoriasis [114, 115], diabetes [116], and Melkersson-Rosenthal syndrome [117]. Patients with teeth-marked tongues often present with clinical symptoms such as loss of appetite, olfactory disturbances related to food, gastric bloating, and diarrhea [118]. Teeth marks are associated with three obesity-related conditions: obstructive sleep apnea [119], nocturnal intermittent hypoxia, and snoring [120]. Prickles appear on the tip or edges of the tongue and can be observed in conditions such as appendicitis, tumors, kidney diseases, gastrointestinal disorders, and COVID-19 [50, 121]. The normal tongue shape is oval, but there are also six other classifications: square, rectangular, round, acute triangular, obtuse triangular, and hammer-shaped [122]. Some clinical findings [11, 26] indicate that a round tongue is associated with gastritis, an obtuse triangular tongue is linked to hyperthyroidism, and a square tongue is related to coronary heart disease or portal hypertension. To further advance the research, multi-instance [103, 123] and fine-grained quantification approaches [121, 124] have been proposed to detect different subtypes within visual representations, including shape, location, number, severity, and their combinations and variations.

*Coating*. In recent years, research on microbial flora has gained significant attention, leading to efforts that integrate tongue characteristics with oral microbiomes as potential diagnostic biomarkers for various diseases [125], including insomnia [126], gastric cancer [127, 128], pneumonia [129, 130], geographic tongue [131]. Consequently, investigations into the characteristics of the tongue coating have emerged to enable more nuanced pathological classifications. The research on tongue coating primarily revolves around the identification of key features such as color, texture, coverage distribution, coverage area, and papillary density [47, 132]. These attributes are of significant importance in determining the prognosis and progression of various diseases.

*Sublingual veins*. The features of the sublingual veins can be used to identify various diseases, including Chronic Venous Insufficiency [133], lung and breast cancer [134]. Current research primarily focuses on assessing disease severity through quantitative indicators of sublingual veins, including color, shape, texture, and geometry (such as length, width, aspect ratio, area, minor half-distance, circular area, circular area ratio, aspect area, and aspect area ratio), as well as the degree of venous dilation (including blood stasis and tortuosity) and the number of ecchymoses [24, 36, 135, 136]. Additionally, some studies have introduced the use of a Hidden Markov Model (HMM) [137] to characterize spectral correlations and inter-band variability, applied to extract sublingual veins from hyperspectral tongue images [27]. Alternatively, near-infrared spectroscopy is used to directly measure the sublingual veins, exploring their relationship with central venous oxygen saturation [138].

*Pathological location*. Tongue image alignment is a fundamental issue in TD, as it involves the mapping of points or sub-regions between different tongue appearances [139]. The reflective points on the tongue are closely related to the occurrence and development of both organic and functional diseases. The tongue surface typically presents various appearances based on the locations of spots [28]. For example, the tip of the tongue is relatively redder and smoother compared to other parts [140], while the area near the tongue root is often covered by coatings of various colors [141]. In clinical practice, the tongue is commonly categorized into four distinct regions: the tip, margin, body, and root [142]. These features are typically distributed across different visual areas and are often accompanied by complex noise [37]. However, in computational research, the delineation of tongue regions is highly unpredictable. As a result, any predefined [18, 140] or fixed [105, 141] method for tongue positioning is vulnerable to distortions due to tongue deformation. Thus, establishing a dense and flexible mapping system is crucial. Several methods, including mapping integration, optical flow techniques, and DL approaches, have been proposed for more accurate regional segmentation, which facilitates further quantitative medical diagnosis.

### Tongue image aided diagnosis

Clinical disease diagnosis relies heavily on the practitioners ability to collect and analyze both physiological and pathological data from patients, ultimately arriving at interpretable conclusions. Current research in the objective assessment of TD primarily focuses on serious diseases, chronic conditions, and infectious diseases. The research primarily focuses on the association analysis between tongue features and diseases [33, 45, 59], as well as early disease screening [43, 143] and prognostic prediction [17, 101, 144] based on physiological, pathological, and biological mechanisms [17, 43, 101, 143, 144]. Developing a systematic approach to using TD for non-invasive early screening of diseases presents significant challenges. It requires not only a deep understanding of existing clinical and prior research but also the ability to establish connections between precise computerized visual perception, physiological mechanisms, and biological pathways.

In clinical practice, a single disease may be associated with multiple states of the tongue. Consequently, multi-label and multi-task models [145–147] have been employed in disease detection studies to enhance the diversity of tongue features. However, these data often suffer from unclear category boundaries. What’s more. A single visual modality is insufficient for fully capturing the disease-related information, prompting the exploration of multimodal approaches to address gaps in pathological data. Commonly used modalities include saliva, endoscopic examination results, fecal antigen diagnostic tests, serological tests, and medical imaging techniques [29, 34, 59, 140, 148–150].

## Challenges and opportunities for CTIA in digital medicine

While CTIA holds great promise, its clinical adoption faces challenges related to standardization, data quality, and integration with other diagnostic tools. Future research should focus on establishing universal guidelines for image acquisition, preprocessing, and feature extraction. Additionally, integrating CTIA with other non-invasive technologies such as saliva testing or wearable sensors could create a comprehensive health monitoring platform, making it a powerful tool for early disease detection and personalized medicine. This section will discuss some of the challenges that have been neglected in related research and explore potential future directions.

### Perception technology

#### Tongue perception within the spatiotemporal domain

Currently, two-dimensional visible-light digital tongue imaging devices are the primary method of perception in research, yet they fall short in providing multidimensional and temporal insights. To extend spatial dimensions, three-dimensional (3D) imaging technologies are essential, with stereo reconstruction systems based on stereoscopic vision and structured light techniques being widely applied. Optical 3D shape measurement technology, in particular, has become well-established in the study of stereoscopic tongue imaging. Additionally, analyzing either long-duration video streams [52–54] or sequential images captured [55–57] at different intervals enables researchers to explore dynamic variations in tongue features over time. Notably, techniques such as hyperspectral imaging, ultrasound, and infrared thermography have been introduced to offer alternative visual perspectives, though studies in these areas are still limited. Looking forward, extremely-weak magnetie functional imaging may present a new visual modality, potentially adding valuable biological insights to tongue research.

In essence, both spatial static imaging and temporal dynamic imaging serve as complementary methods, using tongue images to reveal the physiological and pathological states of the body. Combining dynamic and static information not only demonstrates that the proposed system can perform in vivo detection, but also has the potential to enhance its recognition capabilities [25]. Whereas, from the perspective of feature space, increasing dimensional information does not always enhance an algorithm’s classification accuracy. Particularly with larger datasets, the added computational redundancy can introduce more noise, significantly increasing time complexity and potentially degrading performance. A more effective solution lies in developing a systematic visual feature optimization strategy. By selecting and extracting the most discriminative key information, this approach minimizes redundancy and computational cost while maximizing performance. This direction holds not only theoretical significance but also offers valuable insights for practical applications.

#### Unified data collection standards

In recent years, various digital tongue diagnostic devices and image analysis systems have been widely applied in clinical settings. Their primary advantage lies in the ability to capture high-fidelity images, providing a reliable data foundation for subsequent analysis. However, significant variability remains in the reproducibility of these devices. Differences in models, structural designs, operational methods, and parameter settings lack standardization. Additionally, the absence of clear requirements for critical factors such as lighting conditions and image resolution limits the comparability and reproducibility of diagnostic results. Combined with incomplete diagnostic standards, these issues hinder the broader adoption and application of such technologies in clinical practice.

Furthermore, tongue image data is sourced from diverse origins, and the methods for evaluating data quality vary widely, lacking consistent attribute descriptions and standardized benchmarks. This situation affects not only the standardization and comparability of the data but also introduces potential biases into algorithm development and evaluation. Establishing clear benchmarks and evaluation standards can effectively enhance the reliability of algorithms and systems, reduce unnecessary biases, and provide more robust support for research and applications in the field of TD.

More importantly, the lack of standardized image descriptors significantly hinders the progress toward objective pathological diagnosis. Addressing this issue requires the establishment of a dedicated discipline to scientifically interpret the physiological and pathological implications of tongue images and to develop a systematic pathological classification framework. This would not only provide theoretical support for the CTIA but also advance its deeper application and development in sub-health monitoring and pathological diagnosis.

#### Public datasets and benchmarks

The construction of tongue image databases often relies heavily on clinical resources and requires ethical review, making it challenging to fully publicize the data. However, non-clinically collected data and de-identified image data can provide valuable elements for specific research purposes. While some research teams have acquired tongue diagnostic images under standardized conditions, a universally accepted large-scale standardized database within the industry has yet to be established.

The construction of a standardized tongue image database presents several significant challenges, including data imbalance, the complexity of disease progression patterns, and the necessity for fine-grained descriptions of disease features. Current datasets frequently lack pathologically annotated characteristics that extend beyond the common features of the tongue body and sublingual veins. Furthermore, multi-label, multi-center, and multi-modal datasets remain underdeveloped due to the inherent difficulties associated with their collection, processing, and management. Different collection environments, such as natural, controlled, and mixed settings, along with heterogeneous data sources from various origins, have yet to be systematically quantified or integrated into the design of these datasets.

Furthermore, most existing datasets focus on general tongue characteristics without addressing interpretable pathological features and their clinical mappings. As a result, a comprehensive, high-quality, ethically compliant, and standardized medical dataset is essential in the medical field, as it facilitates precise and reproducible research, deepens understanding of disease, and fosters innovation in diagnostic methodologies [151, 152]. This also enables further adaptation to complex and diverse environments, addressing the data challenges associated with remote healthcare and health monitoring [153].

### Analysis methods

#### Feature quantification

Currently, most research on tongue images primarily focuses on investigating the presence or absence of specific features [154]. However, clinical diagnosis often requires the identification of fine-grained characteristics of tongue images based on objective signals, subjective perceptions, and prior knowledge. Many practitioners emphasize detailed features, such as quantity, morphology, and location, as critical information for disease diagnosis. Unfortunately, these details are rarely documented in medical records, leading to their neglect by many non-medical researchers. This situation highlights a gap in the complementary understanding, explanation, and integration between clinical practitioners and algorithm engineers regarding the objective recognition of TD. Future work can build upon existing recognition efforts to achieve quantitative studies of features through multi-task parallelization, multi-instance analysis, and inter-class partitioning.

#### Deepening and expanding application scenarios

Current research primarily focuses on association analysis between tongue images and diseases, as well as early screening for diseases based on initial feature changes. However, a review of these studies reveals that their depth remains largely confined to simplistic mapping relationships. In reality, more complex efforts should be emphasized, including the exploration of visually based complex mapping relationships, multimodal mappings, and the integration of visual and mechanistic approaches in image and physiological research. Such deepening work is essential for establishing a new paradigm for studying the mechanisms underlying tongue images.

The tongue, with its rich individual characteristics, serves as a valuable biometric feature for identity recognition through its dynamic changes [52]. Additionally, tongue movements are intricately linked to brain functionality, providing a unique and reliable basis for identity verification [155]. It is important to note that, as a non-invasive method of physical examination, TD offers unique advantages in various fields, including health monitoring, chronic disease management, nutritional status assessment, and personalized medicine.

#### Other emerging trends for TD

In the future, leveraging the complementarity and consistency of information across various modalities, alongside automated pattern discovery from unlabelled data, will ensure the stable development of pattern analysis and perceptual computing technologies at their source. Complementary modalities such as microbiome data, spectroscopy, voice, scent, pulse waves, facial visual features, dynamic trajectories, and clinical information can collectively create a comprehensive feature space encompassing diverse types and dimensions.

Despite the advances in visual analysis, there are still shortcomings in understanding and interpreting visual information. With the recent developments in large language models, integrating descriptive issues with outputs from multimodal and knowledge graph approaches has become a primary method for feature description. Incorporating knowledge into pattern recognition and understanding, and applying the results to decision-making and planning, represents a form of advanced cognition that can help mitigate the issue of perceptual illusions [156, 157]. Furthermore, combining visual information from TD and facial analysis with multi-turn active guided questioning, daily dietary records, and medical history interpretation is gradually emerging as a viable new solution. In this technological transformation, TD can also rejuvenate and find new relevance.

Overall, while these challenges underscore the exciting opportunities for advancing CTIA, they also highlight its current limitations. Specifically, issues such as inconsistent acquisition standards, limited and imbalanced datasets, and the interpretability gap between algorithmic outputs and clinical practice remain major barriers to translation. Recognizing these constraints is critical, as it allows researchers to set realistic expectations and to develop targeted strategies that address both the technical and clinical dimensions of CTIA.

## Conclusion

As a non-invasive digital biomarker, tongue imaging not only broadens the scope of medical imaging technologies but also offers novel opportunities for early disease detection and continuous health monitoring. Its integration has the potential to play an increasingly important role in precision medicine. In this review, we synthesized prior work on computerized tongue image analysis (CTIA) by categorizing the research process into two main dimensions: perception and analysis. The perception component encompasses image acquisition devices and systems, data preprocessing, and dataset construction, whereas the analysis component involves feature extraction, anomaly detection, and tongue image–aided diagnosis. Within this taxonomy, we provided a systematic overview of recent advances and identified key research trends that can inform future development.

Despite the promise of CTIA, several important limitations remain. First, the reproducibility of results is hindered by the lack of standardized protocols for image acquisition, preprocessing, and annotation. Second, the available datasets are often limited in scale, imbalanced across disease categories, and inconsistently labeled, which constrains the generalizability of current models. Third, the interpretability of deep learning–based approaches remains a major barrier for clinical translation, as clinicians require transparent and explainable diagnostic evidence. Finally, the integration of CTIA into clinical workflows faces practical challenges, including device heterogeneity, variable environmental conditions, and the need for ethical safeguards in data collection and use.

In light of these limitations, future research should prioritize the establishment of unified acquisition standards, the construction of large-scale annotated databases, and the development of interpretable machine learning frameworks. Additionally, multimodal integration with complementary non-invasive diagnostic tools−such as saliva assays or wearable sensors−may create more robust health monitoring platforms. By addressing these challenges, CTIA may progressively evolve into a valuable adjunct for early disease screening, chronic disease management, and personalized healthcare delivery.

This review acknowledges several inherent limitations. Its narrow focus on computerized tongueue image analysis precluded coverage of all digital biomarker technologies. Although a comprehensive literature search was conducted, the potential for selection bias persists, as some relevant studies may have been omitted due to inconsistent database retrieval strategies or quality assessment exclusions. Heterogeneity in acquisition protocols, datasets, and evaluation metrics further complicates direct comparison across studies. Finally, the rapidly evolving nature of AI and medical imaging means this review may not reflect the very latest advances by the time of publication.

## Additional file

Supplementary file 1.

## Data Availability

No datasets were generated or analysed during the current study.
