# Supplementary material for: Computerized tongue image analysis for non-invasive disease screening: a review
Source: Chin Med. 2025 Nov 21;20:196. doi: 10.1186/s13020-025-01242-7 (PMC12636213; doi:10.1186/s13020-025-01242-7)
Supplement: Supplementary file 1 [file 13020_2025_1242_MOESM1_ESM.zip › Chinese Medicine_appendix/appendix.pdf]

Digital Biomarkers for Non-invasive Disease Screening using Computerized Tongue Image Analysis: A Review(appendices)

Huangbo Lin[1,2], Zhihan Ning[3], Chenglong Zhang[1], Shaoyang Men[2], David Zhang[1*]

[1*]School of Data Science, The Chinese University of Hong Kong, Shenzhen, 2001 Longxiang Boulevard, Shenzhen, 518172, Guangdong, China.

[2]School of Medical Information Engineering, Guangzhou University of Chinese Medicine, 232 Waihuandong Road, Guangzhou, 510006, Guangdong, China.

[3]School of Science and Engineering, The Chinese University of Hong Kong, Shenzhen, 2001 Longxiang Boulevard, Shenzhen, 518172, Guangdong, China.

*Corresponding author(s). E-mail(s): davidzhang@cuhk.edu.cn; Contributing authors: huangbolam@gmail.com; zhihanning@link.cuhk.edu.cn; zhangchl@cuhk.edu.cn; shaoyang.men@gzucm.edu.cn;

A Data augmentation

Data augmentation is a key strategy for improving model performance in medical image analysis. It serves as a regularization technique in supervised learning by applying transformations to the input data that maintain the essential class-related information, ensuring the image's interpretability is preserved[1].

In the field of medical image analysis, obtaining sufficiently large and representative training datasets can be challenging due to the complexities of data acquisition and annotation. Data augmentation techniques address this limitation by transforming existing data or synthesizing new samples, thereby expanding the training dataset

and effectively enhancing the model's generalization capabilities. The primary goals of data augmentation are to boost the volume, quality, and diversity of training data.

Traditional augmentation methods include rotation, flipping, scaling, cropping, color transformation, noise addition, kernel filtering, and kernel perturbation[2, 3]. In addition, with the advancement of DL, novel operations have been introduced, such as image erasure techniques like Cutout and GridMask; multi-image blending methods like Mixup, CutMix, RICAP, and CutBlur; and single-image mixing techniques such as LocalAugment, SelfAugmentation, and SelfMix[4, 5]. These innovative approaches aim to further enhance the diversity and robustness of the training data, ultimately contributing to improved performance in medical image analysis tasks.

While data augmentation techniques have been widely applied in tasks such as segmentation and detection, yielding significant performance improvements, they also face several challenges. These challenges include generating more realistic and diverse medical image data and selecting appropriate data augmentation strategies tailored to specific tasks. Notably, research on generative data augmentation[6] is relatively scarce in the realm of CTIA. In addition, meta-learning techniques can be employed to guide the data augmentation process, further enhancing its effectiveness.

B Dataset construction

Table 1 Display of relevant public datasets.

Ref.	Name	Content	Url
[7]	BioHit	The dataset has 300 tongue images with a size of 576*768, and Manual segmentations were used as the ground truth.	https://github.com/BioHit/TongeImageDataset
[8]	FDV-SV	The dataset was collected from 385 male and female patients with different medical conditions and was manually annotated by lingual and sublingual vein segmentation, with detailed attributes such as width and thickness. All images were taken by non-contact imaging equipment with dimensions 3024 × 4072, and the ages of the different subjects ranged from 25 to 83 years old.	https://github.com/echobear313/FDUVEIN
[9]	FDU/SHUTCM	Acquired 281 images of tongue cracks in JPG image format in a standard environment	https://github.com/pengjianqiang/FDU-TC
[10]	PaddlePaddle dataset-neo	A multi-label dataset containing tongue color, tongue coating, thick and thin, and greasy coating. There are 178 tongue pictures.	https://aistudio.baidu.com/datasetdetail/196398
[11]	Annotated dataset tongue images	668 Tongue Images Collected from Elderly Patients in a Tertiary Hospital in Shanghai, China, and Manually Labeled by Expert Panel.	https://ieee-dataport.org/open-access/annotated-dataset-tongue-images

With the rapid advancements in computer vision, application-focused research continues to evolve, particularly in the medical field, which offers a vast array of resources, including images, texts, and videos. However, a significant challenge lies in effectively leveraging and organizing this data to enhance applications for indexing, retrieval, organization, and user interaction[12]. Unlike general visual domain research, medical data poses unique challenges such as privacy concerns, incomplete datasets, semantic inconsistencies, the standardization of heterogeneous data, effective data management and maintenance, and the development of multi-center databases. In particular, research on tongue diagnosis typically requires the provision of ethical approval. Therefore, acquiring a comprehensive dataset that is both balanced and encompasses all potential scenarios remains a formidable challenge in practical applications. Moreover,

the data distribution in the real world typically follows a long-tail pattern, characterized by an exponential decrease in the number of samples per category from the head to the tail[13–15].

Moreover, for many learning tasks, the cost of collecting labeled data is high due to the inherent need for specialized expertise. This is perhaps best illustrated in medical tasks, where measurements require expensive machinery, and labels are the result of time-consuming analyses conducted by multiple human experts[1]. Such a process makes it challenging to ensure the consistency and accuracy of manual labeling[16]. In open environments, data collection often relies on smart devices or other non-standardized equipment, which makes the data quality vulnerable to external factors such as fluctuations in lighting, differences in handling techniques, and variations in hardware parameters. These factors can cause significant fluctuations in image quality and feature representation, hindering subsequent analysis and modeling. While standardized data collection can be implemented in specific settings, such as private medical clinics, the lack of uniform collection protocols and rigorous quality control often results in smaller datasets with questionable professionalism and reliability. These challenges contribute to issues such as sample imbalance, insufficient diversity, high feature sparsity, and significant data quality variability, which limit the depth and scope of objective tongue diagnosis research. To address these issues, it is essential to optimize collection protocols, enhance data diversity through data augmentation techniques, and introduce cross-device color correction and feature standardization methods to improve data quality throughout the entire collection and processing pipeline. Additionally, during model training, techniques such as sample resampling, re-weighting, cost-sensitive learning, ensemble learning, class-balancing strategies, and feature selection can help mitigate the inherent limitations of the data, ultimately improving the quality and practical value of objective tongue diagnosis research.

Although the public datasets listed in Table 1 predominantly focus on research in segmentation and feature recognition, addressing certain extant challenges, the majority of datasets still lack comprehensive attribute descriptions, such as scenario, scale, hierarchy, accuracy, and diversity. In feature-related studies, there is a deficiency in datasets constructed specifically for pathological conditions, aside from those addressing tongue surface and sublingual vascular features. Furthermore, the construction of multi-label, multi-center, and multi-modal datasets remains unaddressed due to inherent challenges in collection, processing, and management. Different scenarios, including datasets collected in natural environments, standard environmental datasets, mixed datasets, and multi-source heterogeneous datasets, have not been quantitatively considered. More importantly, the majority of datasets focus on general features without addressing the more refined, pathologically interpretable characteristics and their mapping relationships. Therefore, a comprehensive, high-quality, ethically compliant, and standardized medical dataset is essential in the medical field, as it facilitates accurate, reliable, and generalizable research, thereby advancing medical research, deepening the understanding of diseases, and developing innovative diagnostic methods.

C Evaluation Metrics

Evaluation metrics play a pivotal role in assessing the performance of CTIA models. Typical CTIA tasks include 1) *classification* (e.g., disease detection) and 2) *dense prediction* (e.g., tongue segmentation and tongue crack detection).

Generally, classification tasks are the key topic in CTIA, and we provide a detailed analysis of popular classification metrics. Classification metrics can be categorized into *deterministic-based* and *probabilistic-based* metrics. Deterministic-based metrics consider discrete classification outcomes (e.g., predicted class labels), while probabilistic-based metrics take into account the predicted probabilities for each class for evaluation.

C.1 Deterministic-Based Metrics

Deterministic-based metrics include accuracy, precision, recall (sensitivity), F_β score, geometric mean (G-mean), Matthews correlation coefficient (MCC), etc., where F_β score, G-mean, and MCC can generally be used to evaluate both balanced and imbalanced data.

Accuracy. As (1), accuracy measures the overall correctness of predictions.

$$\text{Accuracy} = \frac{\text{TP} + \text{TN}}{\text{TP} + \text{TN} + \text{FP} + \text{FN}}, \tag{1}$$

where TP, TN, FP, and FN stand for true positive, ture negative, false positive, and false negative, respectively. Accuracy may be misleading for imbalanced datasets, as it does not reflect class-specific performance.

Precision. As (2), precision evaluates the proportion of correctly predicted positive cases among all predicted positives

$$\text{Precision} = \frac{\text{TP}}{\text{TP} + \text{FP}} \tag{2}$$

Recall. As (3), recall quantifies the proportion of actual positive cases correctly identified.

$$\text{Recall} = \text{Sensitivity} = \frac{\text{TP}}{\text{TP} + \text{FN}} \tag{3}$$

F_β Score. As (4), F_β score combines precision and recall into a single measure by using β to control the relative importance of recall compared to precision. When $\beta = 1$, (4) denote a popular metric known as F_1 score.

$$F1 = \frac{(1 + \beta^2) \cdot \text{Precision} \cdot \text{Recall}}{\beta^2 \cdot \text{Precision} + \text{Recall}} \tag{4}$$

G-mean. As (5), G-mean calculates the geometric mean of sensitivity and specificity and can be used for both balanced and imbalanced data.

$$\text{G-mean} = \sqrt{\text{Sensitivity} \cdot \text{Specificity}} \tag{5}$$

MCC. As (6), MCC provides a balanced measure that considers true positives, true negatives, false positives, and false negatives. Additionally, it ranges from -1 to 1, which is different from the aforementioned metrics.

$$\mathrm{MCC} = \frac{\mathrm{TP} \cdot \mathrm{TN} - \mathrm{FP} \cdot \mathrm{FN}}{\sqrt{(\mathrm{TP}+\mathrm{FP})(\mathrm{TP}+\mathrm{FN})(\mathrm{TN}+\mathrm{FP})(\mathrm{TN}+\mathrm{FN})}} \tag{6}$$

C.2 Probabilistic-Based Metrics

Probabilistic-based metrics include AUROC, AUPRC, mean squared error (MSE), mean absolute error (MAE), etc., where AUROC and AUPRC can generally be used to evaluate imbalanced data.

AUROC. AUROC denotes the area under the receiver operating characteristic curve, calculated by plotting the TP rate (y-axis) versus the FP rate (x-axis) for all prediction thresholds. Alternatively, it measures the probability that a randomly chosen positive instance is ranked higher than a randomly chosen negative instance by the classifier, as shown in (7).

$$\mathrm{AUROC} = \frac{\sum_{i \in \mathcal{S}^+} \sum_{j \in \mathcal{S}^-} \mathbb{I}(\mathbf{x}_i > \mathbf{x}_j)}{|\mathcal{S}^+| \cdot |\mathcal{S}^-|}, \tag{7}$$

where $\mathcal{S}^+$ denotes positive data, $\mathcal{S}^-$ denotes negative data, and $\mathbb{I}$ is the indicator function that equals 1 when $\mathbf{x}_i > \mathbf{x}_j$ and 0 otherwise.

AUPRC. AUPRC denotes the area under the precision-recall curve, calculated by plotting the recall rate (y-axis) versus the precision rate (x-axis) for all prediction thresholds. One practical formula for approximating AUPRC is using the trapezoidal rule, which numerically estimates the area under the curve by summing the areas of trapezoids between consecutive points on the precision-recall curve as (8).

$$\mathrm{AUPRC} \approx \sum_{i=1}^{K-1} \frac{(r_{i+1} - r_i) \cdot (p_{i+1} + p_i)}{2}, \tag{8}$$

where r_i and p_i are the recall and precision values at the ith threshold.

MSE. As (9), MSE measures the average squared difference between predicted labels and ground truth.

$$\mathrm{MAE} = \frac{1}{N} \sum_{i=1}^{N} (\hat{y}_i - y_i)^2 \tag{9}$$

MAE. As (10), MAE measures the average absolute error of a set of probabilistic predictions.

$$\mathrm{MAE} = \frac{1}{N} \sum_{i=1}^{N} |\hat{y}_i - y_i| \tag{10}$$

It is worth noting that there are other classification metrics, such as cross-entropy loss, Hamming loss, and multi-label metrics. Moreover, pixel accuracy (PA), mean Intersection over Union (mIoU), dice coefficient, boundary F_1 score, etc., are popular

performance metrics for dense prediction tasks such as tongue segmentaion and tongue crack detection. Considering the page limit, additional performance metrics can be checked in ML surveys such as [17–20].

When establishing a dataset and its evaluation metrics, it is essential to consider the implications of the loss function, particularly in the context of medical imaging, where false negatives (FN) often outnumber false positives (FP) due to significant class imbalance issues. Many researchers have attempted to address these challenges. For instance, Chen et al.[21] developed an algorithm that adaptively optimizes hyperparameters in loss functions, improving performance in imbalanced scenarios. Similarly, Ning et al.[22] systematically tackled data imbalance issues by introducing the Balanced Evolutionary Semi-Stacking (BESS) method for disease detection in partially labeled imbalanced (PLI) datasets. They also proposed advanced frameworks such as the Sparse Projection Infinite Selection Ensemble (SPISE) [23] and the Evolutionary Hierarchical Classifier (FEHC) [24], which address challenges in multi-class incremental learning (MCIL) by enhancing both class balance and learning efficiency.

References

[1] Berthelot, D. *et al.* Mixmatch: A holistic approach to semi-supervised learning. *Advances in Neural Information Processing Systems* **32** (2019).

[2] Yan, J. *et al.* Tongue crack recognition using segmentation based deep learning. *Scientific Reports* **13**, 511 (2023).

[3] Hu, Y. *et al.* Automatic construction of chinese herbal prescriptions from tongue images using cnns and auxiliary latent therapy topics. *IEEE Transactions on Cybernetics* **51**, 708–721 (2019).

[4] Mumuni, A. & Mumuni, F. Data augmentation: A comprehensive survey of modern approaches. *Array* **16**, 100258 (2022).

[5] Xu, M., Yoon, S., Fuentes, A. & Park, D. S. A comprehensive survey of image augmentation techniques for deep learning. *Pattern Recognition* **137**, 109347 (2023).

[6] Ruan, Q. *et al.* An efficient tongue segmentation model based on u-net framework. *International Journal of Pattern Recognition and Artificial Intelligence* **35**, 2154035 (2021).

[7] BioHit. Tongueimagedataset. https://github.com/BioHit/TongeImageDataset (2014).

[8] Xiong, Q. *et al.* *Multi-scale generative adversarial network for automatic sublingual vein segmentation*, 851–856 (IEEE, 2020).

[9] Li, X. *et al.* *Automatic tongue image segmentation for real-time remote diagnosis*, 409–414 (IEEE, 2019).

[10] PaddlePaddle. Tongue dataset. https://aistudio.baidu.com/datasetdetail/196398.

[11] Chunlei Tang, X. S. Y. X., Dan Shi. An annotated dataset of tongue images. https://ieee-dataport.org/open-access/annotated-dataset-tongue-images.

[12] Deng, J. *et al.* *Imagenet: A large-scale hierarchical image database*, 248–255 (Ieee, 2009).

[13] Van Horn, G. *et al.* *The inaturalist species classification and detection dataset*, 8769–8778 (2018).

[14] Liu, Z. *et al.* Open long-tailed recognition in a dynamic world. *IEEE Transactions on Pattern Analysis and Machine Intelligence* **46**, 1836–1851 (2022).

[15] Yang, Z. *et al.* *Proco: Prototype-aware contrastive learning for long-tailed medical image classification*, 173–182 (Springer, 2022).

[16] Li, J. *et al.* A multi-step approach for tongue image classification in patients with diabetes. *Computers in Biology and Medicine* **149**, 105935 (2022).

[17] Baştanlar, Y. & Özuysal, M. Introduction to machine learning. *miRNomics: MicroRNA Biology and Computational Analysis* 105–128 (2014).

[18] Jiao, Y. & Du, P. Performance measures in evaluating machine learning based bioinformatics predictors for classifications. *Quantitative Biology* **4**, 320–330 (2016).

[19] Padilla, R., Netto, S. L. & Da Silva, E. A. *A survey on performance metrics for object-detection algorithms*, 237–242 (IEEE, 2020).

[20] Müller, D., Soto-Rey, I. & Kramer, F. Towards a guideline for evaluation metrics in medical image segmentation. *BMC Research Notes* **15**, 210 (2022).

[21] Chen, Y. *et al.* Adaptive region-specific loss for improved medical image segmentation. *IEEE Transactions on Pattern Analysis and Machine Intelligence* **45**, 13408–13421 (2023).

[22] Ning, Z., Ye, Z., Jiang, Z. & Zhang, D. Bess: Balanced evolutionary semi-stacking for disease detection using partially labeled imbalanced data. *Information Sciences* **594**, 233–248 (2022).

[23] Ning, Z., Jiang, Z. & Zhang, D. Sparse projection infinite selection ensemble for imbalanced classification. *Knowledge-Based Systems* **262**, 110246 (2023).

[24] Ning, Z., Jiang, Z. & Zhang, D. To combat multiclass imbalanced problems by aggregating evolutionary hierarchical classifiers. *IEEE Transactions on Neural Networks and Learning Systems* (2024).
